# Comparison of year-of-exam- and age-matched estimates of heritability in the Framingham Heart Study data

**DOI:** 10.1186/1471-2156-4-S1-S36

**Published:** 2003-12-31

**Authors:** Rasika A Mathias, Marie-Hélène Roy-Gagnon, Cristina M Justice, George J Papanicolaou, Yu Ti Fan, Elizabeth W Pugh, Alexander F Wilson

**Affiliations:** 1 Genometrics Section, National Human Genome Research Institute, National Institutes of Health, 333 Cassell Drive, Baltimore, Maryland, USA; 2 Center for Inherited Disease Research, Johns Hopkins University School of Medicine, Baltimore, Maryland, USA

## Abstract

Several different approaches can be used to examine generational and temporal trends in family studies. The measurement of offspring and parents can be made over a short period of time with parents and offspring having quite different ages, or measurements can be made at the same ages but with decades between parent and offspring measures. A third approach, used in the Framingham Heart Study, has repeated examinations across a broad range of age and time, and provides a unique opportunity to compare these approaches. Parents and offspring were matched both on (year of exam) and on age. Heritability estimates for systolic blood pressure, body mass index, height, weight, cholesterol, and glucose were obtained by regressing offspring on midparent values with and without adjustment for age. Higher estimates of heritability were obtained for age-matched than for year-of-exam-matched data for all traits considered. For most traits, estimates of the heritability of the change over time (slope) of the trait were near zero. These results suggest that the optimal design to identify genetic effects in traits with large age-related effects may be to measure parents and offspring at similar ages and not to rely on age-adjustment or longitudinal measures to account for these temporal effects.

## Background

At least three different approaches can be used to examine generational (age) and temporal trends in family studies. In the first, members of different generations are measured over a short period of time (year-of-exam-matched). In the second, observations can be made in the parental generation and then decades later in their offspring with no intervening measures (age-matched). In the third approach, serial observations over time are made contemporaneously in each generation. This approach can be used to adjust for temporal trends and generational differences, but it requires serial observations lasting for generations. The Framingham Heart Study, with repeated examinations across a broad range of age and time in parents and offspring, provides a unique opportunity to compare these different approaches.

If offspring exams are denoted as *o*_*i *_and original cohort (parental) exams as *p*_*j*_, where *i *and *j *represent the year of exam, and *n *and *m *denote the sequence number of the offspring and parent exams, respectively, the combination of offspring (*o*_*i*_) and parental (*p*_*j*_) exams can be represented as a matrix ***D***, such that



where the diagonal elements (*i *= *j*) represent cross-sectional studies matched on closest year of exam and the off-diagonal elements (*i *≠ *j*) represent studies mismatched on year of exam. Offspring can also be matched with their parents on closest age. In this situation, *i *and *j *represent the ages of the offspring and the parents, respectively. When *i *= *j*, the offspring and parents are matched on the exam at their closest age, and when (*i *≠ *j*) the elements of the matrix represent mismatched exams. The availability of serial and contemporaneous measures in both parents and offspring also allows construction of longitudinal phenotypes, such as average and change over time (slope).

In this study, estimates of heritability for measures of systolic blood pressure (SBP), body mass index (BMI), height, weight, cholesterol, and glucose levels were compared in matched and mismatched combinations of offspring and parent exams to determine: 1) whether heritability differs between matched and mismatched exams; 2) whether adjustment for age or other covariates affects estimates of heritability; 3) whether heritability differs between year-of-exam-matched-matched and age data; and 4) whether longitudinal phenotypes provide higher estimates of heritability than estimates from either year-of-exam- or age-matched data.

## Methods

Data sets were created combining measures from the original cohort members and the offspring cohort matching on year of exam or age. For the year-of-exam-matched data, each offspring exam was paired with a corresponding parent exam as indicated in Atwood et al. [1]. Offspring exams for 1971, 1979, 1984, and 1988 were *o*_1_, *o*_2_, *o*_3_, and *o*_4_; parent exams for 1966, 1979, 1983, and 1987 were *p*_10_, *p*_16_, *p*_18_, and *p*_20 _[2]. For the age-matched data, the average age was calculated for each sibship in the offspring exam, and the parent exam was selected so that the parental age was within a one-year interval of the mean offspring age. Because the size of the first offspring exam (*o*_1_) was small, only offspring exams *o*_2_, *o*_3_, *o*_4_, and *o*_5 _were used. Thus, 4 matched and 12 mismatched data sets were created for each matching scheme.

Each data set was analyzed to determine estimates and standard errors of trait heritability. Heritability was estimated using three different regression models: regression of offspring on midparent, of offspring on father, and of offspring on mother. The mean and variance of the estimates of heritability were calculated over the matched exams (diagonal elements) and compared to those calculated over the mismatched exams (off-diagonal elements). The heritability of the average and slope of the trait across the exams was also calculated. Adjustment for covariates was performed by first regressing out the effect of the covariates on the trait in a separate regression including parent and offspring data from all exams. Simple linear age adjustment was used to adjust for age as a covariate. Treatment for high blood pressure was included as a covariate for SBP.

## Results

The results of the estimates of heritability determined from the regression of offspring-on-midparent for age-matched data are illustrated in Table 1 for SBP. Averages and standard deviations over the diagonal and the off-diagonal elements for all traits are presented in Table 2 for all traits. Comparison of the differences of the means of the diagonal and off-diagonal elements provide a measure of the consistency of the estimates of heritability over matched and unmatched exams. Comparisons were also made between estimates of heritability for data unadjusted and adjusted for age, and between year-of-exam- and age-matched data. Table 3 presents mean estimates of heritability for the average trait measure and slope of each trait measure for year-of-exam- and age-matched data. Analyses performed, but not shown, included estimates of heritability based on regression of offspring on father and mother separately and adjustment for covariates other than age.

### SBP

In general, the average estimate of heritability of the matched exams was similar to those of the mismatched exams, regardless of whether the exams were matched on year of exam or age (Tables 1 and 2). There was, however, a substantial difference between the means of the year-of-exam- and the age-matched data sets (0.15 ± 0.03 vs. 0.39 ± 0.06, Table 2). Adjusting for age reduced the heritability to 0.12 ± 0.02 and 0.26 ± 0.05 for year-of-exam- and age-matched exams, respectively. Adjusting for either hypertension medication or age lowered the estimates of heritability in both matching schemes. Overall, none of the adjustments performed removed the difference in heritability seen between the matching schemes.

### BMI, height, and weight

Similarly, there was little difference between means for diagonal and off-diagonal elements for BMI (Table 2). Adjusting for age made little difference in the estimates of the heritability for year-of-exam-matched or age-matched data. There was however, a substantial difference between the means for the year-of-exam- and age-matched data (0.39 ± 0.08 and 0.55 ± 0.22, respectively), although the confidence intervals were large. Estimates of heritability for height were similar for the diagonal and off-diagonal elements, for matching scheme and age adjustment. The estimates of the heritability for weight for the diagonals and off-diagonals were similar within exam, but the estimates for the age-matched exams were higher than those for the year-of-exam-matched data. Adjustment for age made little difference on estimates for both height and weight.

### Cholesterol and glucose

Matching could only be performed on age for cholesterol. The average heritability for the four matched data exams was 0.40 ± 0.05, and estimates were similar in the mismatched exams. Estimates of heritability for glucose were near zero in the year-of-exam-matched data. Age-matched data had higher estimates, but their confidence intervals overlapped zero.

### Longitudinal measures

The estimates of heritability obtained using the trait averages were similar to those from the year-of-exam- and age-matched data sets for most traits (Table 3). Estimates of heritability of change over time (i.e., slopes of traits) however, were close to zero for most traits. Nearly all had confidence intervals overlapping zero. Only the slopes for BMI and height for the age-matched data had estimates of heritability above 0.10 (0.28 ± 0.18 and 0.15 ± 0.18, respectively). Descriptive statistics for the average slope of the regression of each trait over time are presented in Table 4. Only the estimate of the slope for height had a confidence interval that was close to zero.

## Discussion and Conclusions

On average, there was little difference between the diagonal and off-diagonal estimates in either the year-of-exam- or age-matched data. Adjustment for age reduced the estimate of the heritability of SBP, but had little impact on other phenotypes. The estimates of heritability for age-matched data were consistently higher than for year-of-exam-matched data for SBP, height, weight, and BMI. In general, estimates of heritability were higher from regression of offspring on mother than regression of offspring on father (data not shown). And finally, the use of the slope of the trait (a longitudinal measure), resulted in substantially lower estimates of heritability (near zero) than the estimates of heritability for either the year-of-exam- or age-matched data, or the average estimates of heritability.

These results suggest that the optimal design to identify genetic effects in traits with large age-related effects, such as blood pressure, may be to measure parents and offspring at similar ages and not to rely on age adjustment or longitudinal measures to account for these differences.

## Figures and Tables

**Table 1:** Estimates of heritability (± SE) for SBP for age-matched exams.

Offspring Exam	Parent Exam			
	
	*p*_*i*2_	*p*_*i*3_	*p*_*i*4_	*p*_*i*5_
*o*_2_	0.39 ± 0.05	0.35 ± 0.04	0.36 ± 0.03	0.31 ± 0.03
*o*_3_	0.43 ± 0.05	0.41 ± 0.04	0.40 ± 0.03	0.34 ± 0.03
*o*_4_	0.49 ± 0.05	0.49 ± 0.04	0.45 ± 0.04	0.39 ± 0.04
*o*_5_	0.34 ± 0.06	0.38 ± 0.05	0.37 ± 0.04	0.32 ± 0.04

**Table 2:** Mean (± SD) of the estimates of heritability for unadjusted and age-adjusted traits for year-of-exam- and age-matched data.

		Year-of-exam-matched		Age-matched	
		
	Trait	Unadjusted	Age Adjusted	Unadjusted	Age Adjusted
SBP:	Diagonal	0.15 ± 0.03	0.12 ± 0.02	0.39 ± 0.06	0.26 ± 0.05
	Off-diagonal	0.16 ± 0.11	0.12 ± 0.09	0.39 ± 0.06	0.26 ± 0.04
BMI:	Diagonal	0.39 ± 0.08	0.37 ± 0.08	0.55 ± 0.22	0.54 ± 0.22
	Off-diagonal	0.38 ± 0.06	0.37 ± 0.06	0.53 ± 0.27	0.55 ± 0.27
Height:	Diagonal	0.70 ± 0.04	0.70 ± 0.05	0.80 ± 0.08	0.77 ± 0.13
	Off-diagonal	0.70 ± 0.04	0.70 ± 0.04	0.76 ± 0.07	0.74 ± 0.10
Weight:	Diagonal	0.38 ± 0.10	0.38 ± 0.10	0.51 ± 0.08	0.52 ± 0.03
	Off-diagonal	0.39 ± 0.08	0.39 ± 0.08	0.48 ± 0.05	0.49 ± 0.05
Cholesterol:	Diagonal	-^A^	-	0.40 ± 0.05	0.37 ± 0.07
	Off-diagonal	-	-	0.37 ± 0.10	0.34 ± 0.11
Glucose:	Diagonal	0.02 ± 0.04	0.02 ± 0.04	0.10 ± 0.11	0.06 ± 0.10
	Off-diagonal	0.05 ± 0.05	0.04 ± 0.05	0.04 ± 0.15	0.002 ± 0.14

^A^-, not done.

**Table 3:** Mean (± SE) of the estimate of the heritability of the average and slope of the trait for year-of-exam- and age-matched data.

Trait	Trait Average		Trait Slope	
	
	Exam-Matched	Age-Matched	Exam-Matched	Age-Matched
SBP	0.25 ± 0.03	0.44 ± 0.03	-0.01 ± 0.03	-0.10 ± 0.06
BMI	0.38 ± 0.05	0.49 ± 0.06	0.07 ± 0.05	0.28 ± 0.18
Height	0.73 ± 0.05	0.76 ± 0.06	0.09 ± 0.08	0.15 ± 0.18
Weight	0.44 ± 0.06	0.56 ± 0.05	0.06 ± 0.05	0.07 ± 0.08
Cholesterol	-^A^	0.51 ± 0.04	-	0.06 ± 0.07
Glucose	0.08 ± 0.02	0.17 ± 0.04	0.01 ± 0.02	0.07 ± 0.08

^A^-, not done.

**Table 4:** Mean slope (± SE) and 95% confidence intervals (CI) for each trait for all offspring and parent exams combined

Trait	Year-of-exam-matched		Age-matched	
	
	Mean Slope	95% CI	Mean Slope	95% CI
SBP	2.30 ± 0.14	(2.02, 2.57)	1.74 ± 0.13	(1.49, 1.98)
BMI	0.25 ± 0.02	(0.21, 0.29)	0.46 ± 0.02	(0.42, 0.50)
Height	-0.11 ± 0.01	(-0.13, -0.09)	-0.05 ± 0.01	(-0.06, -0.04)
Weight	0.77 ± 0.13	(0.51, 1.03)	2.30 ± 0.11	(2.09, 2.51)
Cholesterol	-^A^	-	3.82 ± 0.29	(3.26, 4.38)
Glucose	1.70 ± 0.27	(1.16, 2.23)	0.76 ± 0.17	(0.42, 1.10)

^A^-, not done.
